# Assessing the Impact of a Gluten-Free Diet on Celiac Disease Symptoms in Children: A Comprehensive Review

**DOI:** 10.7759/cureus.69086

**Published:** 2024-09-10

**Authors:** Hooria Sarwar, Hema Manvi Koneru, Mohit Sinha, Pakeeza Tarar, Rafik Maged, Venkata Varshitha Bandi, Iana Malasevskaia

**Affiliations:** 1 Psychiatry, California Institute of Behavioral Neurosciences & Psychology, Fairfield, USA; 2 Internal Medicine, California Institute of Behavioral Neurosciences & Psychology, Fairfield, USA; 3 Research and Development, California Institute of Behavioral Neurosciences & Psychology, Fairfield, USA

**Keywords:** qol, quality of life, resolution, children, celiac disease, gfd, gluten free

## Abstract

Celiac disease, a serious autoimmune disease, is triggered by the ingestion of gluten. It is associated with many gastrointestinal and extraintestinal symptoms. The cornerstone of treatment is a strict gluten-free diet (GFD). This paper collected studies that were screened between the 15th and 25th of June 2024 and were searched for from many databases and registers, including PubMed, Medline, ClinicalTrials.gov, Cochrane Library, Europe PMC, and EBSCO Open Dissertations. We have included the 12 most relevant studies that examined the effects of GFD adherence among pediatric patients with celiac disease. Evidence suggests that a GFD caused notable improvements in liver function, growth metrics, and quality of life indices. Extraintestinal symptoms such as cardiac dysfunctions and obstructive sleep apnea also showed compelling improvement. We conclude that there are substantial advantages of a GFD in children with celiac disease and call for the need for personal nutritional support to address nutritional deficiencies and long-term studies and comprehensive strategies to optimize treatment outcomes and improve the quality of life for affected children.

## Introduction and background

Celiac disease is a severe autoimmune condition stimulated by gluten, a protein found in wheat, rye, and barley, that results in injury of the small intestine lining [1,2]. The prevalence of celiac disease has been estimated to be approximately 1%, but only 30% of sufferers are accurately diagnosed [2]. Children need to be diagnosed early to prevent long-term complications such as growth retardation, neurological symptoms, and increased risk for the development of other autoimmune diseases [1].

In children, the clinical presentation of celiac disease varies greatly and can be divided into intestinal (e.g., diarrhea and abdominal pain) and extraintestinal (e.g., growth impairment) manifestations [3], nutritional deficiencies, neurologic disorders, dermatologic conditions, bone mineral loss, hepatic dysfunction [4], cardiac abnormalities [5], and alterations in body composition. These late sequelae can be avoided with timely diagnosis and effective management.

The first step in diagnosing celiac disease is serological testing for specific antibodies, including anti-endomysial antibodies (EMA) or anti-tissue transglutaminase (tTG) [6].

Serological testing is a primary diagnostic tool that is used to test for celiac disease. Some of the serological tests used in celiac diagnosis include, but are not limited to, endomysial antibodies IgA-EMA and IgG-EMA, IgA/IgG anti-gliadin antibody (AGA), and anti-tissue-transglutaminase (tTG) antibodies. A positive result through a serological test usually prompts a confirmatory small intestinal biopsy that reveals characteristic signs of villous atrophy, crypt hyperplasia, and increased intraepithelial lymphocytes. Genetic testing for *HLA-DQ2* and *HLA-DQ8* may also support the diagnosis in ambiguous cases [6]. One major treatment plan for celiac disease is a lifelong gluten-free diet (GFD). Following GFD leads to symptom relief, mucosal healing, and prevention of recurrence of disease-related complications [7]. There is a well-established relationship between the presence of celiac disease-specific antibodies and the severity and prognosis of the disease [8]. However, efficacy differs among patients on GFD therapy due to the challenge of adhering strictly to it, as gluten is found in many food products.

This narrative review highlights significant gaps in managing pediatric celiac disease, including difficulties with maintaining strict adherence to a GFD and variability in treatment efficacy. It underscores the need for more effective adherence strategies and a holistic approach to managing both intestinal and extraintestinal symptoms. Addressing these gaps is crucial for improving the understanding and treatment of celiac disease in children. The rationale for this review is driven by the rising prevalence of celiac disease among pediatric populations and the vital role of dietary management in improving health outcomes. Given the diverse manifestations of the disease, both gastrointestinal and extraintestinal, a comprehensive review of existing literature is essential to synthesize findings on the effects of adherence to a GFD. This review aims to equip clinicians and caregivers with evidence-based insights to refine dietary management strategies and enhance the quality of life for affected children.

## Review

A comprehensive search for relevant literature was undertaken to identify studies investigating the efficacy of a GFD in managing celiac disease in the pediatric population. Six electronic databases were systematically searched (June 15-25, 2024): PubMed, Medline, ClinicalTrials.gov, Cochrane Library (CENTRAL), Europe PMC, EBSCO Open Dissertations, and ScienceDirect. The search strategy employed a combination of keywords and MeSH terms related to GFD, celiac disease, pediatric population, and treatment outcomes. Table 1 presents the detailed search strategy.

**Table 1:** Search strategy

Search Strategy	Databases/Registers	Number of Studies Before and After Filters	Filters Applied
(("Gluten-Free Diet" OR "Gluten free") AND ("Celiac disease") AND (Child OR Adolescent OR Pediatric OR Pediatrics) AND ("Treatment Outcome" OR "Symptom Relief" OR "Recovery of Function" OR Improvement OR "Remission Induction")	PubMed	736/57	Full text, Clinical Study, Clinical Trial, Equivalence Trial, Observational Study, Randomized Controlled Trial, Adolescent: Child: birth-18 years
("Diet, Gluten-Free"(Mesh) OR "Diet, Gluten-Free/trends"(Mesh)) AND ("Celiac Disease"(Mesh)) AND ("Child"(Mesh) OR "Adolescent"(Mesh)) AND ("Treatment Outcome"(Mesh) OR "Recovery of Function"(Mesh))	Medline	123/16	Full text, Clinical Study, Clinical Trial, Equivalence Trial, Observational Study, Randomized Controlled Trial, Child: birth-18 years
Intervention: Gluten-free diet, Disease: Celiac Disease in children	ClinicalTrials.gov	11/3	Completed studies | Child (birth - 17) | Studies with results
Date Run: 07/07/2024 17:29:38 Comment: ID Search Hits #1 ("Gluten-Free Diet" OR "Gluten free"):ti,ab,kw 725 #2 MeSH descriptor: (Diet, Gluten-Free) explode all trees 165 #3 #1 OR #2 725 #4 ("Celiac disease"):ti,ab,kw 1091 #5 MeSH descriptor: (Celiac Disease) explode all trees 498 #6 #4 OR #5 1091 #7 (Child OR Adolescent OR Pediatric OR Pediatrics):ti,ab,kw 307323 #8 MeSH descriptor: (Child) explode all trees 82330 #9 MeSH descriptor: (Adolescent) explode all trees 137486 #10 MeSH descriptor: (Pediatrics) this term only 947 #11 #7 OR #8 OR #9 OR #10 307323 #12 ("Treatment Outcome" OR "Symptom Relief" OR "Recovery of Function" OR Improvement OR "Remission Induction"):ab 186368 #13 MeSH descriptor: (Remission Induction) this term only 4880 #14 MeSH descriptor: (Treatment Outcome) explode all trees 203082 #15 #12 OR #13 OR #14 356638 #16 #3 AND #6 AND #11 AND #15 31	Cochrane Library (CENTRAL)	31/31	Filters Language: English Clinical Trials
("Gluten-Free Diet" OR "Gluten free") AND "Celiac disease") AND (Child OR Adolescent OR Pediatric OR Pediatrics) AND ("Treatment Outcome" OR "Symptom Relief" OR "Recovery of Function" OR Improvement OR "Remission Induction") AND (observational study OR interventional study OR "clinical trial") NOT (review OR Meta-analysis)	Europe PMC	17/13	Full text or link to full text research articles
("Gluten-Free Diet" OR "Gluten free") AND ("Celiac disease") AND (Child OR Adolescent OR Pediatric OR Pediatrics) AND ("Treatment Outcome" OR "Symptom Relief" OR "Recovery of Function" OR Improvement OR "Remission Induction") AND (''observational study'' OR ''interventional study'' OR "clinical trial" OR ''RCT'') NOT (review OR ''Meta-analysis'')	EBSCO Open Dissertations	59/59	No filters applied
Title, abstract, keywords: ( "Gluten-free") AND "Celiac disease") AND (Child OR Adolescent OR Pediatric) AND ("Treatment Outcome" OR "Symptom Relief" OR Improvement OR "Remission Induction")	Science Direct	10/6	Article type: research article

The search across all six electronic databases identified a total of 172 articles. To manage and efficiently screen this volume of literature, a reference management software program, Rayyan app® (Qatar Computing Research Institute, Ar-Rayyan, Qatar) [9] was utilized. Following duplicate removal (n=69), 103 articles remained for further evaluation. Titles and abstracts were then screened according to the predefined inclusion criteria (Table 2), resulting in the exclusion of 96 articles. The final number of studies included in this review is seven from the initial search and five from the citation search, including a total of 12 studies for future analysis.

**Table 2:** Inclusion and exclusion criteria RCT: randomized controlled trials

Category	Inclusion Criteria	Exclusion Criteria
Population	Children & adolescents (0-18 yrs) with gastrointestinal &/or extraintestinal symptoms	Adults
Intervention	Primary intervention: Gluten-free diet	Combined dietary interventions/treatments
Comparison	Standard diet, placebo, or no intervention	-
Outcomes	Outcomes related to the resolution or improvement of symptoms and/or extraintestinal symptoms -/+ Biochemical/laboratory outcomes	Studies that do not measure relevant outcomes related to the resolution or improvement of symptoms
Study Design	RCTs, cohort studies, case-control studies, observational studies	Case reports, case series, editorials, reviews, commentaries, no comparison group/baseline data
Publication Type	Peer-reviewed articles, theses, dissertations, trials (completed with results)	Non-peer-reviewed articles, conference abstracts/posters (without full data), trials (non-completed or completed without results)
Language	English	Other than English
Time Frame	Last 10 years (January 1, 2014 to June 25, 2024)	Published before 2014 and after June 25, 2024
Subjects	Human	Animal

Several studies have explored the impact of GFD on various health aspects. The findings from these studies are summarized in Table 3.

**Table 3:** Summary of the included studies GFD: gluten-free diet; MPI: myocardial performance index; OSA: obstructive sleep apnea; cIMT: carotid intima-media thickness; QoL: quality of life; PSQ: pediatric sleep questionnaire; Height-S: Height-SDS; FFM: fat-free mass; MM: muscle mass; TBW: total body water; BCM: body cell mass; BMI: body mass index; FTT: failure to thrive; T1DM: type 1 diabetes mellitus

Study	Study Design	Aim of Study	Key Findings
Pedoto et al., 2020 [3]	Prospective observational study	1. Primary aim: assess adherence to GFD in children with celiac disease; 2. Secondary aim: identify factors influencing diet adherence and related outcomes, such as growth and QoL	This study found that diet adherence was excellent in 59.4%, fair in 28.8%, and low in 11.9% of patients. The study concluded that the factors influencing adherence to GF are the parents’ knowledge and awareness of the disease, financial status, and school career (age of the patient), suggesting a negative relationship between compliance and biopsy-sparing approaches
Joshi et al., 2018 [4]	Observational prospective study	To determine the prevalence of celiac disease in patients with cryptogenic cirrhosis and assess the impact of a GFD on liver function in those diagnosed with celiac disease	13.1% of patients with a specific liver condition (cryptogenic cirrhosis) had celiac disease. Comparison of data at presentation and six months: patients with both conditions benefited from a gluten-free diet, showing improvement in blood tests, liver function, and overall health scores. GFD prevents progression to hepatic failure and delays liver transplant
Bolia et al., 2018 [5]	Longitudinal observational analysis	To evaluate the effects of a GFD on cardiac function over one year	After one year on a GFD: Improved heart function (isovolumic relaxation time, deceleration time, p<0.001) in compliant children; lower MPI (0.60 vs. 0.66, p=0.04). Untreated children exhibited significantly (p<0.04) larger left ventricle end-diastolic dimension, reduced left ventricular ejection fraction (p<0.01), and higher MPI ( p≤0.01) compared to healthy controls
Yonis et al., 2021 [10]	Cohort study	To study the effect of GFD adherence on the growth parameters of children with celiac disease	FTT was noted in (26%) and (34.8%) of the GFD-compliant and GFD non-compliant groups, respectively. Difference = μ (GFD COMPLIANCE) - μ (GFD NON-COMPLIANCE). The estimated difference of 0.590 was significant, with a 95% CI for the difference: (-0.837; -0.344). DF = 38. T-Value = -4.84, P-Value = (0.000)
Yerushalmy-Feler, 2018 [11]	Prospective cohort study	To investigate the effect of a GFD on OSA symptoms in children with celiac disease	Celiac disease group: Fewer OSA-related symptoms than controls; significant improvement after six months on a gluten-free diet. Control group: Higher rate of positive Pediatric Sleep Questionnaire (PSQ) scores at both recruitment (33.3% vs. 11.8%, P=0.046) and 6-month follow-up (16.7% vs. 0%, P=0.014). Follow-up improvement: PSQ scores showed improvement in both groups (P<0.001). Greater improvement in the celiac group (0.1 ± 0.09) compared to controls (0.06 ± 0.06, P=0.04)
Demir et al., 2016 [12]	Prospective, observational, cross-sectional study	To analyze carotid intima-media thickness and arterial stiffness as early markers of atherosclerosis in pediatric celiac disease	cIMT was significantly correlated with tTg IgA antibody level, which is the marker of uncontrolled celiac disease (non-adherence to GFD). Lower cIMT in celiac disease with strict gluten adherence suggests that GFD seems to have a beneficial effect on cardiovascular profile. cIMT (mm) in CD with tTg IgA(+) (0.43±0.08) decreased to 0.4±0.05 in CD with tTg IgA (-). P=0.262
Więch et al.,2018 [13]	Case-control study	To assess the impact of a GFD on the body composition of children with celiac disease	After one year of a GFD, celiac disease children showed significantly higher values of FFM (kg) (p= 0.001), MM (kg) (p < 0.001), TBW (L) (p < 0.001), and BCM (kg) (p< 0.001), a significantly higher increase in weight (p= 0.034) and BMI (p= 0.021)
Soliman et al., 2019 [14]	Prospective cohort study	To evaluate the linear growth of children with celiac disease after the first two years on a gluten-free diet	Height-SDS remained either normal or increased in 28/30 children with celiac disease on GFD during the year of treatment (-0.38 ±1.2 to -0.22±1.1), with a positive trend of 0.15±0.4 SDS
Taskin et al., 2022 [15]	Retrospective cohort study	To evaluate factors affecting the prognosis of pediatric celiac disease	Significant improvement in developmental parameters (weight, BMI z-scores, and BMI percentile) was observed in patients adhering to the gluten-free diet at the control visit. Adherence to the GFD is a critical factor for achieving positive intervention outcomes (P<0.002)
Sansotta et al., 2020 [16]	Cohort study	To analyze the GFD impact on growth of children in two different countries	GFD seems to improve nutritional status in American children, decreasing the proportion of overweight (from 17% to 12%) and underweight (only 4%) celiacs. In Italy, there was a slight increase in the proportion of overweight (from 6% to 9%) and especially of underweight (19%) celiac children following the GFD. Of note, 50% of Italian CD children who were underweight on the GFD had a previously normal BMI, and roughly 20% of them were found not to be adherent to the GFD and/or had other comorbidities, which were likely the main determining factors
Franceschi et al., 2024 [17]	Cross-sectional study	Association of GFD with quality of life in youths with type 1 diabetes and celiac disease	The analysis showed that individuals with both T1DM and celiac disease who did not strictly adhere to a GFD had significantly lower overall QoL scores compared to those with only T1DM (p=0.0014)
Kozioł-Kozakowska et al., 2021 [18]	Cohort study	To evaluate one-year follow-up of anthropometric parameters in children and adolescents with celiac disease	While children with celiac disease experienced growth in weight (4kg p<0.01) and height (8cm p<0.01) after one year on a gluten-free diet (GFD), yet there was no significant increase in BMI or BMI percentile after six months (accordingly Δ = 0.08, Δ = −0.42) and 12 months of the study (accordingly Δ = 0.32, Δ = −0.05)

Discussion

The Effect of a GFD on Gastrointestinal Symptoms

The efficacy of a GFD in improving gastrointestinal symptoms is well-documented across several studies. Joshi et al. (2018) [4] evaluated the impact of a GFD on clinical outcomes. They observed improvements in the Child-Pugh score (CPS) initially and after six months of GFD in cases of chronic liver disease with celiac disease. The parameters observed were encephalopathy, ascites, serum albumin, serum bilirubin, and prothrombin time. After six months of GFD, statistically significant improvement was seen in serum albumin levels (p=0.001) and prothrombin time (p=0.001). This study was able to highlight the possibility of delaying or improving liver damage in celiac disease by adhering to GFD [4].

Growth and Quality of Life With a GFD

Pedoto et al. (2020) [3] used a questionnaire to study growth and quality of life in pediatric patients with celiac disease and reported significant impacts of a GFD on growth parameters and quality of life in this patient population. A strong correlation was observed between better compliance and higher quality of life at PGWBI (Psychological General Well-Being Index); this suggests how dietary adherence strongly contributes to the general wellness and QoL, as reported in other studies [3].

Impact on Extraintestinal Manifestations

Bolia et al. (2018) [5] identified subclinical cardiac dysfunction in untreated pediatric celiac disease patients. Adherence to a GFD for one year improved heart function, particularly in compliant children, as evidenced by reduced myocardial performance index (MPI) (0.60 vs. 0.66, p=0.04). Conversely, non-compliant children exhibited larger left ventricular end-diastolic dimension (p<0.04), reduced left ventricular ejection fraction (p<0.01), and higher MPI (p≤0.01) compared to healthy controls [5].

Furthermore, Yerushalmy-Feler et al. (2018) [11] reported a significant association between celiac disease and reduced obstructive sleep apnea (OSA) symptoms in children. Children with celiac disease exhibited a lower prevalence of OSA-related symptoms (11.8% vs. 33.3%) compared to controls at baseline. Following a GFD, the celiac group demonstrated greater improvement in OSA symptoms, as measured by the pediatric sleep questionnaire, compared to controls (0.1 ± 0.09 vs. 0.06 ± 0.06) [11].

Demir et al. (2016) [12] demonstrated in their study that pediatric celiac disease patients who follow strict GFD showed a decrease in cIMT (carotid intima-media thickness). Higher cIMT is correlated with the pathogenesis of lesions of atherosclerosis [19]. Additionally, they observed that cIMT (mm) in celiac disease with positive tissue transglutaminase antibody (tTg IgA+) decreased from 0.43 ± 0.08 to 0.4 ± 0.05 in celiac disease patients with negative tissue transglutaminase antibody (tTg IgA-) (p=0.262). Hence, strict adherence to a GFD seems to have a beneficial effect on atherosclerotic risk factors [12].

Linear Growth and Body Mass Index (BMI)

Więch et al. (2018) [13] investigated body composition changes in children with celiac disease adhering to a GFD for at least one year. Children on a GFD exhibited significantly higher fat-free mass (p=0.001), muscle mass (p<0.001), total body water (p<0.001), and body cell mass (p<0.001) compared to baseline [13]. Additionally, weight (p=0.034) and BMI (p=0.021) increased significantly. These findings suggest that a GFD may positively influence body composition in children with celiac disease [13].

Similarly, Soliman et al. (2019) [14] analyzed the longitudinal impact of a GFD on linear growth in children with celiac disease. Height standard deviation scores (SDS) remained stable or improved in 28 out of 30 children after a year on a GFD (-0.38 ± 1.2 to -0.22 ± 1.1), indicating catch-up growth. These findings suggest that a GFD can positively influence linear growth in children with celiac disease [14].

Possible Confounding Variables

Several key factors, such as adherence level, duration of diet, and other dietary restrictions, could confound the outcomes related to adherence to a GFD in children with celiac disease, as highlighted by the studies included in the review. Pedoto et al. (2020) found that adherence varied significantly, with 59.4% of participants showing excellent adherence [3]. This variability suggests that outcomes such as growth and QoL are closely linked to how well patients follow the GFD. Similarly, Yonis et al. (2021) noted that non-compliance was associated with higher rates of FTT, reinforcing the importance of adherence [10].

Another critical factor is the length of time on a GFD. For instance, Bolia et al. (2018) observed significant improvements in cardiac function after one year on the diet, indicating that longer adherence may yield more substantial health benefits [5]. Shorter follow-up periods may not fully capture the diet's effects. Additionally, the limited sample size in the study by Soliman et al. [14] may weaken the overall strength of the findings.

Additional dietary restrictions can also influence outcomes. Sansotta et al. (2020) noted that some children in Italy who were underweight on a GFD had previously normal BMIs, suggesting that other health conditions or dietary limitations might play a role in their nutritional status [16].

While the studies provide valuable insights into the effects of a GFD, considering these confounding variables is essential for a more nuanced understanding of the findings. Addressing these factors in future research will enhance our interpretation of how adherence to a GFD impacts health outcomes in pediatric patients.

Challenges and Considerations

Taşkın et al. (2022) [15] emphasized the importance of early diagnosis and strict adherence to a GFD in optimizing outcomes for children with CD. However, maintaining long-term GFD compliance presents significant challenges. While Więch et al. (2018) [13] demonstrated improvements in body composition among children adhering to a GFD, concerns regarding nutritional sufficiency persist, highlighting the need for ongoing dietary monitoring and supplementation [13]. These findings underscore the complex interplay between disease management, patient adherence, and long-term health outcomes in children with CD.

Interestingly, GFD compliance is shown to have a different impact on the growth of children in different countries/cultures [16]. Sansotta et al. (2020) observed the effect of a GFD on children living in Italy and the USA. It was observed that GFD seems to improve nutritional status in American children, decreasing the proportion of overweight (from 17% to 12%) and underweight (from 6% to 4%) celiac patients. In Italy, there was a modest increase in the nutritional status of celiac children following a GFD, with the proportion of overweight children rising from 6% to 9% and the proportion of underweight children increasing significantly from 9% to 19%. It is noteworthy that 50% of Italian CD children who became underweight on the GFD had a previously normal BMI. The reasons for these differences remain unclear and may be multifactorial, but it is speculated to stem from different cultural aspects, including the availability of gluten-free food and lifestyle factors [16].

Another study by Franceschi et al. (2024) [17] concluded that children who are diagnosed with celiac disease and type 1 diabetes mellitus (T1DM) report a low QoL when adherent to GFD compared to children who are diagnosed with T1DM only (p=0.0014). The QoL questionnaire inquired about physical well-being, emotional well-being, self-esteem, family, friends, and daily routine. Franceschi et al. inferred "that the restrictive nature of a GFD, combined with typical developmental issues that adolescents have to face and all tasks resulting from managing a lifelong health condition, may impact a child's emotional well-being and exacerbate the subjective perception of living in an unhealthy body" [17].

Furthermore, a cohort study conducted by Kozioł-Kozakowska et al. (2021) [18] found that while children with celiac disease experienced growth in weight (4 kg, p<0.01) and height (8 cm, p<0.01) after one year on a GFD, their nutritional status, as measured by BMI percentile, showed little change. BMI percentiles were calculated after six months (accordingly Δ=0.08, Δ= −0.42) and 12 months of the study (accordingly Δ=0.32, Δ= −0.05). Despite adherence to the GFD, there were limited improvements in nutrient intake, indicating ongoing risks for deficiencies in calcium, iodine, iron, folic acid, and vitamins D, K, and E [18]. These findings emphasize the need for personalized nutritional guidance beyond general GFD recommendations to address the specific dietary needs of individuals with celiac disease.

Comparison With Other Evidence

Kozioł-Kozakowska et al. (2021) [18] found significant improvements in weight and height in children with celiac disease after one year on a GFD, yet only minimal changes in BMI percentiles were observed, indicating limited impact on overall nutritional status. This finding is consistent with broader research on GFD's efficacy and limitations.

Cardo et al. (2021) [20] found that while GFD effectively alleviates gastrointestinal symptoms and promotes growth, it often fails to fully address nutritional deficiencies. This finding is corroborated by Vici et al. (2016) [21], who reported persistent deficiencies in essential nutrients such as calcium, iron, and vitamin D despite adherence to GFD.

Beyond nutritional deficiencies, several ongoing concerns have been identified in the literature. For instance, Wolf et al. (2018) [22] observed that while GFD is beneficial for symptom management, strictly adherent GFD shows a lowered quality of life and psychological well-being in teenagers compared to teenagers who are less vigilant with GFD. This is supported by Rostami-Nejad et al. (2020) [23], who found that GFD duration shows no improvement in anxiety symptoms.

Moreover, Marciniak et al. (2021) [24] highlighted that while GFD helps manage celiac disease symptoms, imbalanced GFD does not address broader health concerns, leading to obesity, type 2 diabetes mellitus, metabolic syndrome, and dyslipidemia. These results highlight that, beyond adhering to a GFD, it is essential to address nutritional deficiencies as well as psychological, developmental, and overall health factors through tailored dietary and medical interventions.

Strengths and Limitations of the Review and Included Studies

This detailed review has benefited from a thorough search of various databases, resulting in a wide array of relevant studies that explore the management of celiac disease through a GFD in pediatric patients. One significant strength of this review is its focus on diverse research designs, which encompass both gastrointestinal and extraintestinal outcomes. This multifaceted approach provides a nuanced understanding of the GFD's impact, particularly in the demographic of children and adolescents that often present unique challenges in dietary management due to higher rates of food intolerance. By addressing both types of outcomes, the review offers valuable insights into the complexities of managing celiac disease in young patients.

However, several limitations are evident in both the review and the included studies. Since this is not a systematic review, there is no formal quality assessment of the studies analyzed, which may affect the reliability of the conclusions drawn. Methodological biases, such as variability in adherence measurement and potential selection bias, may also impair the overall validity of the findings. Additionally, many studies followed participants for only short durations, making it difficult to assess long-term adherence to the GFD and its sustained effects on health outcomes. The small sample sizes in some investigations further limit the generalizability of the results to broader populations. Despite these challenges, this review highlights critical areas for future research, emphasizing the need for standardized methodologies and long-term studies to better understand the efficacy of GFD in managing celiac disease in pediatric patients.

Future Research Directions

We conclude that the most critical areas of investigation relate to the priority of long-term studies in evaluating GFD's effects on growth, development, and general quality of life. Research on dealing with a potential nutrient deficiency is also important to celiac disease management. Furthermore, research into the role of demographic and cultural differences might provide insight into the variation in management outcomes. Additionally, educational programs and support systems tailored for children and their families are the center point of the situation. The progress and psychological well-being of the patients can be better understood by using standardized questionnaires that measure the quality of life of the patients. The inclusion of telehealth and digital tools for dietary education and support goes a long way in making the education and management of disease a lot easier.

## Conclusions

In conclusion, this review underscores the pivotal role of a GFD in managing both gastrointestinal and extraintestinal manifestations of celiac disease in pediatric patients. Our findings reveal that adherence to a GFD significantly enhances growth parameters, quality of life, and various health outcomes, including cardiac function and nutritional status. However, challenges related to long-term adherence and the risk of nutritional deficiencies highlight the necessity for continuous dietary monitoring and tailored nutritional support. Clinicians must prioritize early diagnosis and patient education to ensure optimal adherence to the GFD, thereby improving health outcomes for children with celiac disease.

Looking ahead, future research should focus on the long-term effects of GFD adherence across diverse populations, particularly examining how cultural factors influence dietary compliance and health outcomes. Additionally, there is a need for studies that evaluate the effectiveness of supplementary nutritional interventions to address deficiencies commonly associated with GFD. These insights could significantly impact clinical practice and policy-making, ultimately enhancing the comprehensive management of pediatric celiac disease and ensuring better health trajectories for affected children.
